# Metastatic Temporal Bone Tumor From Renal Cell Carcinoma Coexisting With Sphenoid Ridge Meningioma: A Case Report

**DOI:** 10.7759/cureus.73198

**Published:** 2024-11-07

**Authors:** Yusuke Otsu, Shin Yamashita, Terukazu Kuramoto, Kazuhide Shimamatsu, Motohiro Morioka

**Affiliations:** 1 Neurosurgery, Omuta City Hospital, Omuta, JPN; 2 Diagnostic Pathology, Omuta City Hospital, Omuta, JPN; 3 Neurosurgery, Kurume University, Kurume, JPN

**Keywords:** embolization of the artery feeding the tumor, metastatic bone tumor, middle meningeal artery, renal cell carcinoma, sphenoid ridge meningioma

## Abstract

Metastatic renal cell carcinomas (RCC) of the skull are relatively rare. Here, we present a rare case of a skull tumor due to metastatic RCC coexisting with a sphenoid ridge meningioma. A 69-year-old man was followed up for a sphenoid ridge meningioma. He had undergone a laparoscopic right nephrectomy for RCC 10 years previously. He had a new tumor in the right temporal bone, which rapidly grew with bone destruction within a short period of five months. Both tumors had a common feeding artery, and embolization of the artery feeding the tumor was performed before tumor resection. Intraoperative findings and postoperative imaging of both tumors confirmed total resection. The histopathological results indicated metastatic RCC of the skull and meningothelial meningioma. Systemic radiological examination revealed a metastatic lung tumor, and the patient was transferred to another hospital with a modified Rankin scale (mRS) score of 2 for chemotherapy. The presence of meningioma could have induced the development of metastatic bone tumors via a common feeding artery. It is effective to perform embolization of the artery feeding the tumor before tumor resection.

## Introduction

Renal cell carcinoma (RCC) is the most common form of kidney cancer in adults, accounting for approximately 3% of all adult malignancies. RCC generally has an unpredictable metastatic pathway, and late recurrence is a characteristic of this tumor, which shows a high rate of metastasis to the lungs, lymph nodes, bones, and liver [1]. However, very few cases of skull metastasis have been described [2]. In cases of skull metastasis, it frequently invades the local vascular network by direct extension, while metastatic tumor cells reach the head via hematogenous flow [3]. Some cases of skull metastasis take the form of tumor-to-tumor metastasis, and there have been previous reports of RCC metastasis within meningiomas [4-5]. However, there have been no reports of RCC occurring in the skull apart from a meningioma. Herein, we present a rare case of metastatic RCC of the skull coexisting with a sphenoid ridge meningioma.

## Case presentation

**Figure 1:**
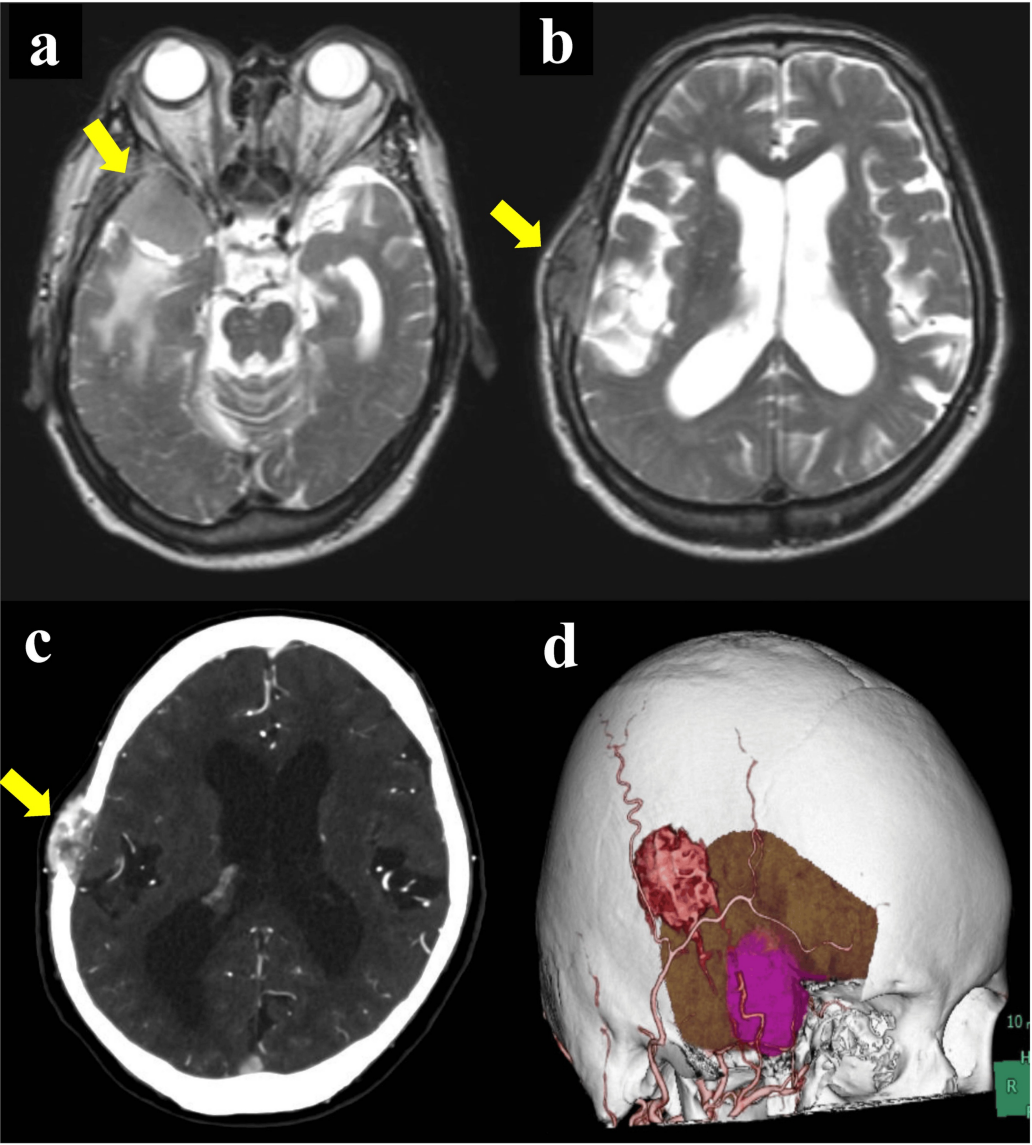
Preoperative image T2-weighted magnetic resonance imaging (MRI) showing an existing right sphenoid ridge meningioma (a, yellow arrow) and a rapidly growing right temporal bone tumor (b, yellow arrow). Computed tomography angiography (CTA) showing an osteolytic plastic lesion with abundant blood supply to the right temporal bone (c, yellow arrow). In the 3D-CT component image of the surgical simulation, the temporal bone tumor (red mass lesion) and sphenoid ridge meningioma (purple mass lesion) are in close proximity, with feeding arteries in the external carotid artery system (d).

The patient was a 69-year-old man undergoing hemodialysis for chronic renal failure after undergoing laparoscopic right nephrectomy for right RCC 10 years ago. Six years after nephrectomy, a right sphenoid ridge meningioma was incidentally found, and the patient underwent follow-up at our hospital. He had further developed a new bone tumor on the right temporal bone five months earlier. The tumor rapidly enlarged, resulting in bone destruction, in a short period of five months only, while the meningioma also showed a tendency to increase simultaneously (Figures 1a-1c). Therefore, we planned to remove both tumors simultaneously. In the preoperative 3D-computed tomography (CT) component image of the surgical simulation, the temporal bone tumor and sphenoid ridge meningioma were in close proximity to feeding arteries from the external carotid artery system (Figure 1d). Furthermore, the two tumors shared a common feeding artery, and we performed embolization of the artery feeding the tumor prior to tumor resection.

**Figure 2:**
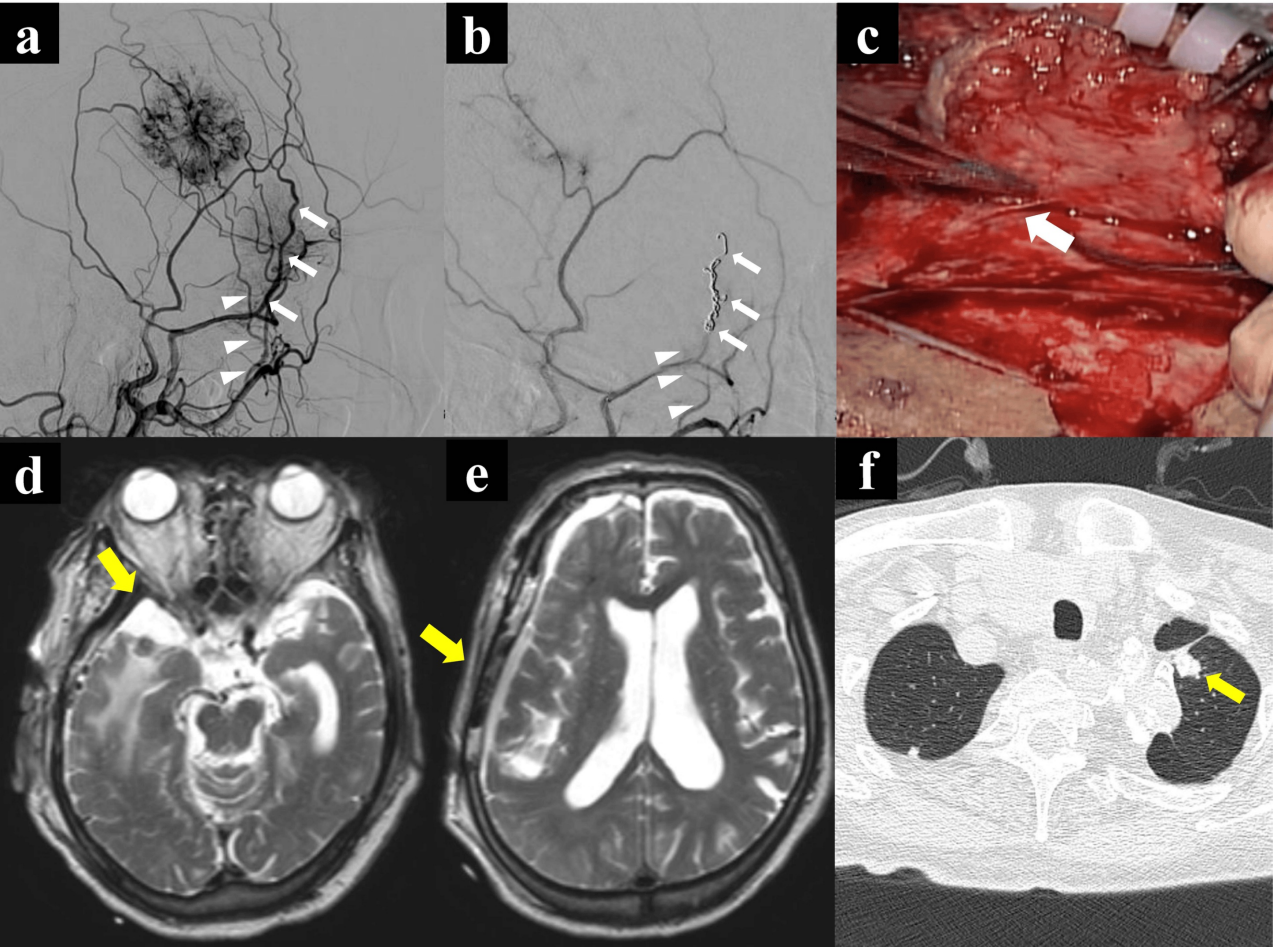
Pre-postoperative digital subtraction angiography (DSA), intraoperative image, and postoperative MRI and CT image DSA of the right internal maxillary artery (IMA) showing that both the right temporal bone tumor and the sphenoid ridge meningioma were supplied with blood from the middle meningeal artery (MMA) anterior branch (a, white arrows). The bone tumor is also supplied by the anterior deep temporal artery (ADTA, white arrowheads). Following preoperative embolization, digital subtraction angiography (DSA) of the right IMA confirmed that the MMA anterior branch was embolized with coils (white arrows), and ADTA was embolized with embosphere microspheres (b, white arrowheads). The temporal bone tumor had blood supply from the MMA during the operation (c, white arrow). Postoperative MRI showed the total removal of sphenoid ridge meningioma (d, yellow allow) and temporal bone tumor (e, yellow arrow). The postoperative systemic radiological examination revealed a metastatic left lung tumor (f, yellow arrow).

Under local anesthesia, the anterior deep temporal artery (ADTA), a feeding artery of the bone tumor, was embolized using embosphere microspheres (Merit Medical Systems, South Jordan, USA). The middle meningeal artery (MMA) anterior branch, a common feeding artery for both tumors, was subsequently embolized using an embosphere and microcoils (Figures 2a-2b). Tumor resection revealed a reddish, elevated bone tumor with a blood supply from the MMA (Figure 2c). The sphenoid ridge meningioma had no adhesions to the internal carotid artery or optic nerve, and Simpson grade 2 tumor resection was performed. Postoperative magnetic resonance imaging (MRI) confirmed the complete removal of the metastatic bone tumor and meningioma (Figures 2d-2e). Systemic radiological examination revealed a metastatic left lung tumor postoperatively (Figure 2f).

**Figure 3:**
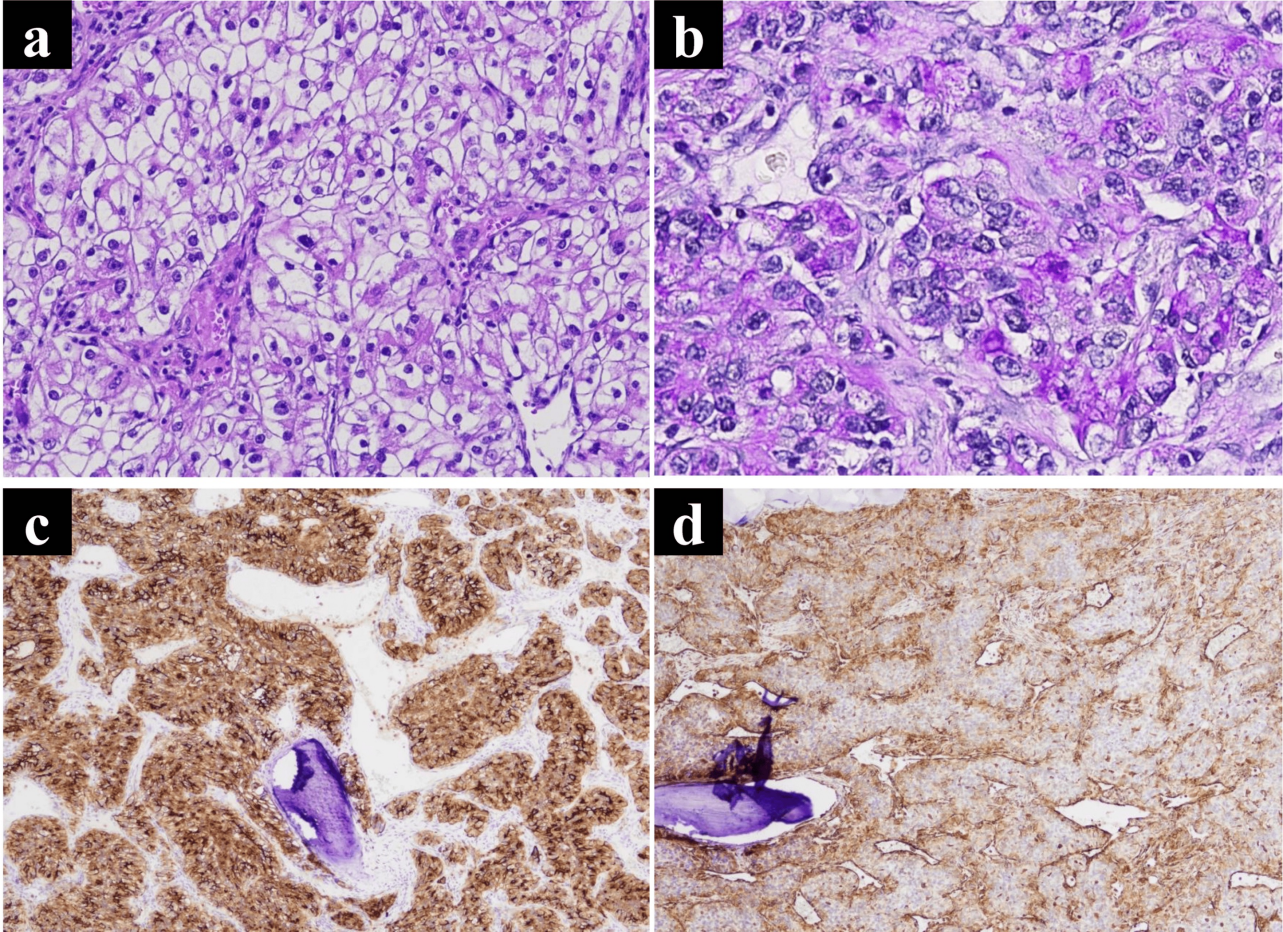
Histological findings of the metastatic bone tumor Hematoxylin and eosin (H&E) staining showing tumor cells composed of abundant clear to eosinophilic cells in a lobular pattern surrounded by a fibrous band (a). Periodic acid-Schiff (PAS) staining was positive in clear cells. (b) Tumor cells were positive for CD10 (c) and vimentin (d).

Histopathological examination of the bone tumors revealed that the tumor cells were composed of abundant clear to eosinophilic cells in a lobular pattern, surrounded by a fibrous band (Figure 3a). Periodic acid-Schiff (PAS) staining was positive for clear cells, while the tumor cells were positive for CD10 and vimentin, resulting in a diagnosis of metastatic clear cell RCC. However, the right sphenoid ridge meningioma was diagnosed as meningothelial meningioma (Figures 3b-3d). The patient was transferred to another hospital with a modified Rankin scale (mRS) score of 2 for chemotherapy. We scheduled regular follow-up head CT scans at the transfer facility, with plans to continue imaging follow-up at our hospital after the patient is discharged.

## Discussion

RCCs commonly metastasize as hematogenous or lymphogenous tumors. In the present case, the RCC metastasized not only to the temporal bone but also to the lungs, and it is unclear whether it metastasized to the temporal bone via lung metastasis or to the lung and temporal bone at the same time. If both tumors occur simultaneously, the metastatic route to the temporal bone may be via Batson’s vertebral venous plexus [6]. However, as skull metastasis itself is rare and clear cell carcinoma often metastasizes to the lungs, RCC is likely to metastasize to the temporal bone via lung metastasis [2,7].

Bone metastases of RCC tend to occur in the spine, ribs, and pelvis, but skull metastases are relatively rare, occurring in only 1.5-6.5% [8]. Metastatic bone tumors often occur when cancer cells enter the bone through feeding arteries, reach the venous sinus, and invade the bone marrow tissue [9]. Meningiomas have a rich vascular supply, which can increase the risk of providing vascular supply and developing environment to other tumors [10]. Although the superficial temporal artery often becomes a feeding artery for metastatic cranial tumors, the MMA may rarely act as a feeding artery [11]. In the present case, a sphenoid ridge meningioma was observed before the metastatic bone tumor developed in the temporal bone. We checked the MMA as a feeding artery of the cranial tumor during the operation. Digital subtraction angiography (DSA) also revealed that the dilated MMA anterior branch was a common feeding artery for meningioma and metastatic cranial tumors, while the preexisting meningioma increased blood flow to the feeding artery, making it susceptible to hematogenous metastasis to the temporal bone via MMA. In this situation, tumor-feeding embolization before tumor resection is effective as the two tumors have an abundant blood supply and a common feeding artery. Preoperative embolization can facilitate surgical resection, which may reduce intraoperative blood loss and surgical time [12].

Histopathologically, there was no RCC metastasis within the meningioma, making it distinct from a collision tumor or tumor-to-tumor metastasis [13-14]. In the present case, only HE and immunohistochemical stainings were performed, and the molecular genetic relationship between the two tumors was therefore unclear. The molecular genetic relationship between the two tumors further could be clarified with comprehensive analyses, such as next-generation sequencing [15].

Although the molecular genetic association between sphenoid ridge meningioma and metastatic bone tumors could not be clearly established in the present case, we cannot deny the possibility of increased blood flow and development of the MMA anterior branch due to the preexisting meningioma. Further studies accumulating more cases are required to understand the pathogenesis.

## Conclusions

The presence of meningioma that increases blood flow in the external carotid artery system might be able to induce the development of metastatic bone tumors via a common feeding artery. It is effective for the coexisting tumors that have a common feeding artery to perform tumor-feeding embolization simultaneously before tumor resection.
